# Proteomics and life-history variability of Endogenous Phospholipases A2 Inhibitors (PLIs) in *Bothrops jararaca* plasma

**DOI:** 10.1371/journal.pone.0295806

**Published:** 2024-02-06

**Authors:** Caroline Serino-Silva, Caroline Fabri Bittencourt Rodrigues, Jackson Gabriel Miyamoto, Daniela Miki Hatakeyama, Victor Koiti Kavazoi, Marisa Maria Teixeira Da Rocha, Aparecida Sadae Tanaka, Alexandre Keiji Tashima, Karen de Morais-Zani, Kathleen Fernandes Grego, Anita Mitico Tanaka-Azevedo

**Affiliations:** 1 Laboratório de Herpetologia, Instituto Butantan, São Paulo, SP, Brazil; 2 Programa de Pós-Graduação Interunidades em Biotecnologia (PPIB—IPT, IBU and USP), Universidade de São Paulo (USP), São Paulo, SP, Brazil; 3 Departamento de Bioquímica, Universidade Federal de São Paulo (UNIFESP), São Paulo, SP, Brazil; Monash University - Malaysia Campus, MALAYSIA

## Abstract

In Brazil, the genus *Bothrops* is responsible for most ophidian accidents. Snake venoms have a wide variety of proteins and peptides exhibiting a broad repertoire of pharmacological and toxic effects that elicit systemic injury and characteristic local effects. The snakes’ natural resistance to envenomation caused by the presence of inhibitory compounds on their plasma have been extensively studied. However, the presence of these inhibitors in different developmental stages is yet to be further discussed. The aim of this study was to evaluate the ontogeny of *Bothrops jararaca* plasma inhibitor composition and, to this end, plasma samples of *B*. *jararaca* were obtained from different developmental stages (neonates, youngs, and adults) and sexes (female and male). SDS-PAGE, Western blotting, affinity chromatography, and mass spectrometry were performed to analyze the protein profile and interaction between *B*. *jararaca* plasma and venom proteins. In addition, the presence of γBjPLI, a PLA_2_ inhibitor previously identified and characterized in *B*. *jararaca* serum, was confirmed by Western blotting. According to our results, 9–17% of plasma proteins were capable of binding to venom proteins in the three developmental stages. The presence of different endogenous inhibitors and, more specifically, different PLA_2_ inhibitor (PLI) classes and antihemorrhagic factors were confirmed in specimens of *B*. *jararaca* from newborn by mass spectrometry. For the first time, the αPLI and βPLI were detected in *B*. *jararaca* plasma, although low or no ontogenetic and sexual correlation were found. The γPLI were more abundant in adult female, than in neonate and young female, but similar to neonate, young and adult male according to the results of mass spectrometry analysis. Our results suggest that there are proteins in the plasma of these animals that can help counteract the effects of self-envenomation from birth.

## 1. Introduction

Snakes are a group of animals that attract human interest because of their psychological and culture influences, their ecological and public health importance, and as a source of molecules with biotechnological potential [1–5]. There are currently 3,848 snake species worldwide (data from www.reptile-database.org), of which about 1,100 venomous snakes are of medical importance [6], responsible for 1.8 to 2.5 million cases of snakebite per year [7–9]. In Brazil, the average number of snakebites per year is approximately 27,000 and most of these accidents are caused by *Bothrops* species [10]. The venom of these snakes is a complex mixture of proteins that induces specific local and systemic effects that can lead to severe complications and death [11]. Although the most effective treatment for snakebite envenomation is the use of antivenom serum, usually composed by polyclonal immunoglobulins purified from large mammals hyperimmunized with snake venom, there are still reports that local effects are not completely neutralized, and patients may be at risk of adverse effects [12–14]. Therefore, countless efforts have been made in the search of treatments that can replace and/or support the use of antivenom serum against snakebite [15, 16]. One of the most important sources for studies has been the blood of animals resistant to snakebite. Such resistance may occur due to coevolutionary pressure between prey and predator (common in mammals), or simply due to the need to protect themselves from their own venom (common in venomous snakes) [17–22]. Moreover, the origin of these molecules is usually related to the evolution of both toxin inhibitors and its targets by gene duplication, gene turnover, or neofunctionalization [23, 24].

Venom inhibitors are molecules that deactivate toxins from the venom before they reach their targets [23]. Natural inhibitors against metalloproteinases, phospholipases A_2_ (PLA_2_), serine proteases, and C-type lectins have already been previously described in animal plasma [21, 23, 25–28]. Specifically, in *Bothrops jararaca* (*B*. *jararaca*) blood, some of these inhibitors were identified, purified and characterized. For example, BJ46a and BJ46b (isoform less abundant), have a molecular mass of 46 kDa and are able to inhibit the hemorrhagic activity of the metalloproteinases atrolysin C and jararhagin (class P-I and class P-III, respectively) [29]; BjI, a protein with two polypeptide sequences of 109 and 139 kDa, has a native molecular mass of more than 1,000 kDa and recognizes thrombin-like enzymes (serine proteases) and inhibits clotting activity [30, 31]; fibrinogen, with a total molecular mass of 372 kDa and three chains of 71, 60, and 55 kDa, was shown to be resistant to clotting by various snake venoms (*B*. *jararaca*, *Crotalus durissus terrificus*, and *Lachesis muta*) [19]; and finally, γBjPLI, a phospholipase A_2_ inhibitor (PLI) with a molecular mass of approximately 20 kDa, inhibits the enzymatic, edematogenic, and myonecrotic activities of PLA_2_ from *Crotalus durissus terrificus* [32].

Ontogenetic changes have been frequently detected in various snake venoms [33–37]. In *B*. *jararaca*, these changes are related to the lethality of the venom and the type of diet, since it feeds on endothermic animals in the adult phase and on ectothermic animals in the young phase [38]. The major components in the venom of this species are the metalloproteinases classes P-I and P-III (more abundant in neonate venom) and serine proteinases (more abundant in adult venom); however, no differences were found in the expression of PLA_2_ between neonate and adult venoms [39, 40].

Although several studies describe ontogenetic differences in the composition of snake venoms, studies on the ontogeny of snakes’ plasma protein composition to ensure natural resistance to self-envenomation are scarce. There are only two works in the literature addressing plasma ontogeny. Gomes et al. (2017) [33] performed a comparative analysis of ontogenetic variation in the expression of transcript profiles of plasma proteins with potential antivenom activity of young and adult *B*. *jararaca* snake. In this transcriptomic analysis, the inhibitor BJ46a showed no ontogenetic difference. On the other hand, a PLI isoform and two protease inhibitors (inter-alpha-trypsin inhibitor and C1 inhibitor) showed higher expression in the adult group [33]. In another study, Morais-Zani et al. (2013) [31] demonstrated the distinct levels of BJ46a (more expressed in adults) and PLI (more expressed in young) in a proteomic analysis of *B*. *jararaca* plasma.

**Fig 1:**
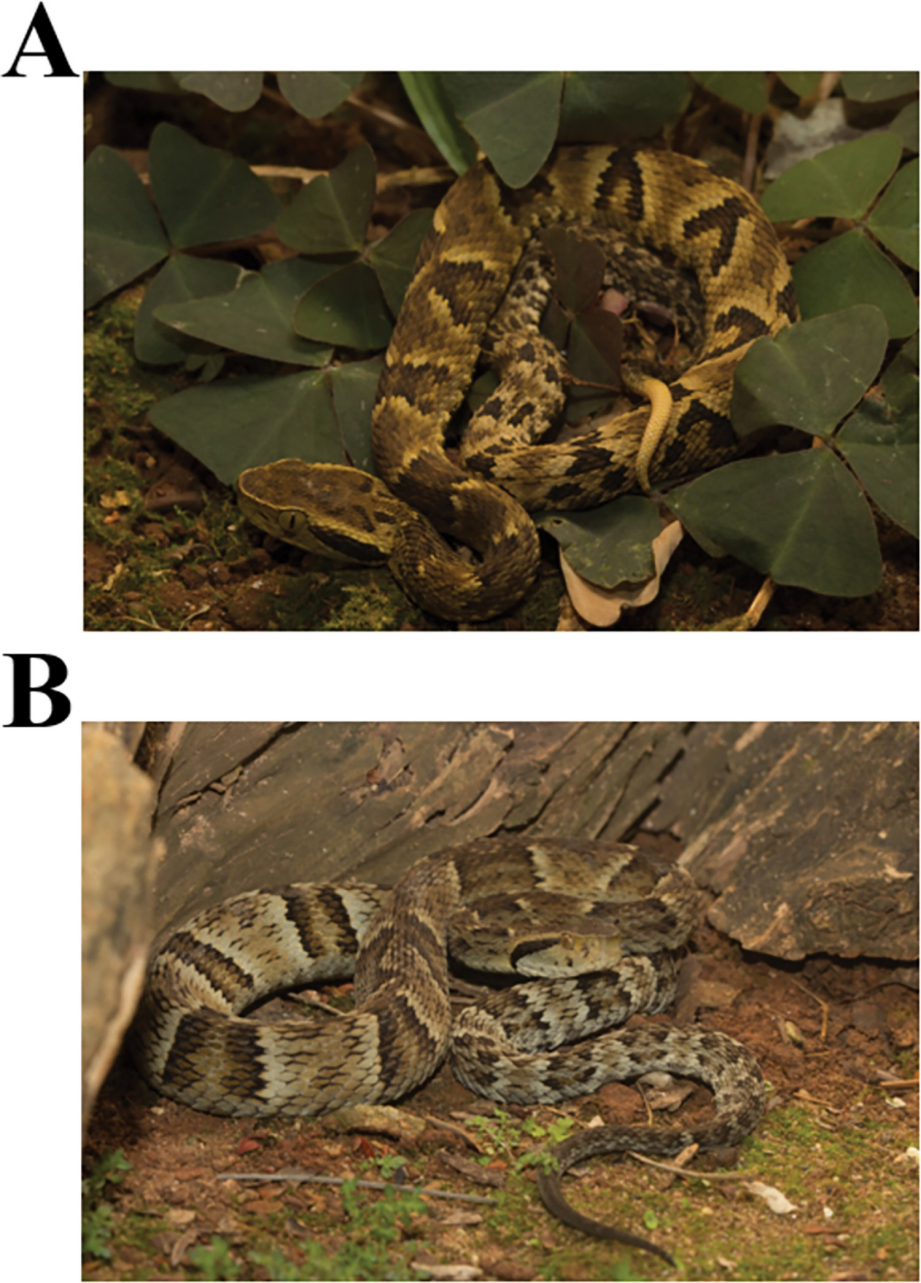
Snakes used in the work: **(A)** Neonate and **(B)** adult *Bothrops jararaca* (Sávio Stefanini Sant’Anna).

Although the hypothesis linking ontogenetic variation in snake venoms to diet is well established, it is important to consider that there are other factors at play [41]. Metabolic processes related to growth, maturation, and physiological transitions may impact the production, turnover, and distribution of proteins [42–44]. Developmental constraints, such as limited resources or genetic factors, may impose limitations on the production or effectiveness of snake venom inhibitors in plasma. Additionally, selective pressures from the environment, as hunting habits, habitats used, prey availability, competition with other species, and specific survival needs at different stages of development, may also influence in the presence or activity of venom inhibitors, and need to be further studied [41–44]. In this context, the aims of this work were to contribute to the knowledge on plasma inhibitors and clarify the ontogenetic differences of plasma proteins with natural resistance to venom by comparing female and male specimens of newborn, young and adult *B*. *jararaca* snakes (**Fig 1**). To this end, analyses of plasma proteins were performed by SDS-PAGE, Western blotting, affinity chromatography, and mass spectrometry, focusing on the proteins that recognize their own crude venom.

## 2. Materials and methods

### 2.1. Animals’ethics statement

Individual *B*. *jararaca* snakes were kept in captivity in the Laboratory of Herpetology at Instituto Butantan and female Balb/C mice were obtained from Rodents Animal Facility at Butantan Institute. All procedures involving animals followed the ethical principles for animal research adopted by the Brazilian Society of Animal Science and the National Brazilian Legislation n° 11.794/08. The blood and venom were collected following strict protocols to minimize stress and suffering. After the *in vivo* tests, mice were euthanized using a CO_2_ chamber, according to ethical parameters approved by the National Council for Control of Animal Experimentation (CONCEA) and the Committee for the Ethical Use of Animals of Instituto Butantan (CEUAIB) (protocol n° 7751060117 and 7598110219).

### 2.2. Snake plasma and venom

All snakes (*B*. *jararaca*) utilized in the present study are kept in captivity in the Laboratory of Herpetology’s snake house (Instituto Butantan, São Paulo, Brazil) in plastic boxes at controlled temperature, in a light/dark 12-hour cycle, with water *ad libitum*. Once a month, the snakes were fed only with small rodents (*Mus musculus* and/or *Rattus norvegicus*) in proportion to their weight (10–20% of the snake’s body weight) [45]. Milked venoms of *B*. *jararaca* from different localities, sex, and age, either from the wild or born in captivity were pooled and underwent a lyophilization process for preservation of its compounds.

According to the snake’s total weight, 1% of the blood volume was collected by caudal venipuncture in tubes containing 0.38% sodium citrate from 90 *B*. *jararaca* specimens of different ages **(Table 1)**. Samples were pooled in six separate groups: neonate female (NF), neonate male (NM), young female (YF), young male (YM), adult female (AF), and adult male (AM). Plasma was obtained by centrifugation at 1,200 x *g* for 15 min and stored at -20°C until use [41].

**Table 1:** Information of the individual plasma used in this study.

	ID	Age (months)	Geographic origin^#^	Sex	Individuals number^&^	Weight (g)	Lenght SVL/TL (cm)
Neonate	NF1	0.06	Araçariguama—SP	F	6	8.6	28/31
	NF2	0.06	Piedade—SP	F	4		
	NF3	0.06	Itapevi—SP	F	5		
	NF4	0.06	Cotia—SP	F	4		
	NF5	0.06	São Bento do Sul—SC	F	4		
	NF6	0.06	Araçariguama—SP	F	4		
	NM1	0.06	Araçariguama—SP	M	8	8.9	28/31
	NM2	0.06	Piedade—SP	M	7		
	NM3	0.06	Itapevi—SP	M	9		
	NM4	0.06	Cotia—SP	M	4		
	NM5	0.06	São Bento do Sul—SC	M	7		
	NM6	0.06	Araçariguama—SP	M	5		
Young	YF1	16	São Bento do Sul -SC	F	-	118	73/81
	YF2	19	São Bento do Sul -SC	F	-	113	67/76
	YF3	20	Machado—MG	F	-	76	57/64
	YF4	20	Machado—MG	F	-	86	59/68
	YF5	20	Juquitiba—SP	F	-	90	66/75
	YF6	20	Cotia—SP	F	-	102	65/73
	YM1	16	Ibiúna—SP	M	-	103	68/79
	YM2	16	São Bento do Sul -SC	M	-	86	64/74
	YM3	19	São Bento do Sul -SC	M	-	66	53/64
	YM4	19	São Bento do Sul -SC	M	-	66	55/65
	YM5	20	Machado—MG	M	-	66	54/63
	YM6	20	Juquitiba—SP	M	-	90	72/83
	YM7	20	Juquitiba—SP	M	-	75	67/78
Adult	AF1	35*	São Bento do Sul -SC	F	-	230	92/104
	AF2	74*	São Franscisco de Paula—RS	F	-	575	112/125
	AF3	54*	Embu das Artes—SP	F	-	680	117/134
	AF4	37*	São Bento do Sul—SC	F	-	470	112/123
	AF5	23*	Mairiporã -SP	F	-	695	122/140
	AM1	42*	Cotia—SP	M	-	175	95/168
	AM2	36*	São Luiz do Paraitinga—SP	M	-	185	86/99
	AM3	25*	Juquitiba—SP	M	-	275	101/113
	AM4	36*	Ibiúna—SP	M	-	230	101/115
	AM5	26*	Tapiraí—SP	M	-	195	92/110

* The age of adults refers to the time they are in captivity.

# The geographic origin of neonates and young specimens refers to the geographic origin from where their mother was captured in the wild, since all these animals were born in captivity.

& "Individuals number" refers to the number of specimens used to form the plasma pool of the neonate group.

### 2.3. Quantification of plasma-venom protein binding

To quantify the protein percentage in snake plasma-venom protein binding, an affinity chromatography technique was utilized. This involved the coupling of venom to a resin and subsequent passage of the plasma through the column. The capture of plasma components with affinity for venom proteins was performed using a protocol modified from Pla, Rodríguez, & Calvete (2017) [46]. One hundred milligrams of CNBr-activated Sepharose Fast-Flow resin were hydrated with 750 μL of 1 mM HCl pH 2.7. The resin was washed with 15 column volumes (CV, 350 μL) of hydration solution and equilibrated with 2 CV of coupling buffer (0.2 M NaHCO_3_ e 0.5 M NaCl pH 8.5). Right after that, resin was incubated with 6 mg of *B*. *jararaca* venom in 1 CV of the coupling buffer for 2 hours on an orbital shaker. After incubation, the affinity column was washed with 10 CV of coupling buffer and blocked with 0.1 M Tris-HCl pH 8.0, overnight on an orbital shaker. After these procedures, the column was washed with 5 CV of the acid wash buffer (0.1 M sodium acetate buffer and 0.5 M NaCl pH 4.0) intercalating with basic wash buffer (0.1 M Tris-HCl and 0.5 M NaCl pH 8.0).

After the column preparation, we proceeded with the affinity chromatography. The column was equilibrated with 10 CV of PBS (140 mM NaCl, 2.6 mM KCl, 10 mM Na_2_HPO_4_, 1.7 mM KH_2_PO_4_, pH 7.4). Three mg of plasma proteins from each pool were diluted in 500 μL of PBS and applied to the column, which was kept on an orbital shaker for 2 hours. After incubation, the unretained proteins were washed with PBS. The bounded proteins were eluted with 10 CV of elution buffer (0.1M glycine-HCl buffer pH 2.7), and the eluate was fractionated at each 2 CV in microtubes with 100 μL of Tris pH 8.8 to neutralize the acid pH. Direct concentration measurement was performed on the NanoDrop™ One (Thermo Fisher Scientific) at 280 nm, with an estimated percent of extinction coefficient (ε1%) of 10. The analysis was performed in triplicate and the data was expressed in mg/mL [47].

### 2.4. Analyzing plasma-venom protein binding

To analyze the plasma protein profile, additional techniques were employed, including electrophoresis and recognition of plasma proteins through venom by Western blotting methodology.

Twenty μg of NF, NM, YF, YM, AF, and AM plasma pools were applied in SDS-PAGE (12%), performed according to Laemmli 1970 [48], in the presence of reducing conditions (β-mercaptoethanol). The gels were electrophoresed at a constant amperage of 20 mA and the samples stained with Coomassie Brilliant Blue G-250. Dual Color Precision Plus Protein Standards (BioRad) was used as a molecular weight standard.

To investigate the presence of venom-interacting molecules present in the plasma of the three life stages, this immune recognition assay method was performed. Twenty micrograms of the samples were separated by 12% SDS-PAGE and then electrotransferred using Trans-Blot Turbo Transfer System (Bio-Rad) onto PVDF membranes, previously equilibrated in transfer buffer (25 mM Tris, 192 mM glycine, 20% ethanol). It was used a constant current of 2.5 A and voltage up to 25 V for 35 min. The membranes were blocked with TBS-milk (10 mM Tris-HCl, 150 mM NaCl, 0.1% Tween 20 and 5% non-fat milk, pH 7.5) overnight at 4°C. The membranes were sensitized with 40 μg of *B*. *jararaca* venom (1:1,000) in TBS-milk for 1 h at 4°C. Afterwards, the membranes were incubated with 1:1,000 antibothropic serum (antivenom from Instituto Butantan produced by horses hyperimmunization with a pool of venom from *B*. *jararaca* (50%), *B*. *jararacussu* (12.5%), *B*. *alternatus* (12.5%), *B*. *moojeni* (12.5%), and *B*. *neuwiedi* (12.5%) for 2 h at room temperature, followed by an incubation with 1:10,000 peroxidase-labeled anti-horse IgG (Sigma) for 2 h at room temperature. After each incubation, the unbound antibodies were washed 3 times with wash buffer (10 mM Tris-HCl, 150 mM NaCl and 0.1% Tween 20, pH 7.5). The immunoreactive bands were visualized using diaminobenzidine (Sigma) and H_2_O_2_, according to the manufacturer’s recommendations [49].

### 2.5. Detection of γBjPLI presence in snake plasma

Initially, female Balb/C (18–22 g) mice were housed in a temperature-controlled room with automatic 12-h light/dark cycle. Food and water were freely available during the study period. Mice were anesthetized with one drop of 1% tetracaine hydrochloride, 0.1% phenylephrine hydrochloride (Allergan) and blood samples (200 μL) were collected by puncture of the orbital plexus to obtain pre-immune serum (negative control). Subsequently, mice were immunized by subcutaneous injection of 10 μg of isolated γBjPLI (purified according to Serino-Silva et al. [32]) per mice, with 50 μg of aluminum hydroxide as adjuvant.

Two additional booster injections were administered every 15 days after the first injection. The sample of immune blood (200 μL) was collected three times by puncture of the orbital plexus also every 15 days after the first immunization. Serum was separated from clots by centrifugation at 1500 x *g* for 15 min, aliquoted, and stored at -20 C. After the experiments, the animals were euthanized using a CO_2_ chamber, according to the ethical parameters described previously.

Mice serum titers were determined by ELISA using 96-well plates coated overnight at 4°C with 5 μg (100 μL) of *B*. *jararaca* serum in carbonate coating buffer (34 mM Na_2_CO_3_ and 15 mM NaHCO_3_, pH 9.6). The plates were then blocked with 200 μL of carbonate coating buffer (pH 9.6) containing 5% fat free milk (CCB-milk) for 2 h at 37°C and incubated with different dilutions of the serum in CCB-milk containing 0.05% Tween 20 (incubation buffer) for 1 h at 37°C. The plates were then washed three times with PBS containing 0.05% Tween 20 (washing buffer) and incubated with peroxidase-conjugated second antibody (anti-mouse IgG) (Sigma) diluted in incubation buffer (1:5,000). After 1 h incubation at 37°C, the plates were washed three times with washing buffer, and the complexes were visualized using 100 μL of ortho-phenylenediamino (1 mg/mL; Sigma) in 0.2 M sodium citrate buffer pH 5.0 containing 0.01% H_2_O_2_, and color increase was stopped by adding 50 μL 30% H_2_SO_4_. Absorbance was recorded at 492 nm using a microplate reader (SpectraMax i3, Molecular Devices). All samples were assayed in triplicates. Results were expressed as mean ± SDM [50].

The Western blotting protocol was carried out as described in section 2.4. The membrane (containing samples from *B*. *jararaca* plasma pools) was incubated with anti-γBjPLI (1:1,000) in an incubation solution (0.5% milk and 0.01% Tween 20) for 1 hour at room temperature. Subsequently, the membrane was incubated with the immunoenzyme conjugate (anti-mouse IgG conjugated with peroxidase, Sigma) diluted 1:10,000 in incubation buffer for 2 hours at room temperature.

### 2.6. Proteomic analysis of plasma of snakes by mass spectrometry

Pools of NF, NM, YF, YM, AF, and AM plasma containing 100 μg of proteins were dissolved in 50 mM NH_4_HCO_3_ and incubated with 0.1% RapiGest surfactant (Waters) at 80°C for 15 min, followed by centrifugation at 2000 x *g* for 3 min; then reduced with 5 mM dithiothreitol (DTT) for 30 min at 60°C, alkylated with 15 mM of iodoacetamide for 30 min in dark at room temperature. Samples were re-incubated with DTT at room temperature for 15 min to prevent excessive alkylation. Trypsin (Promega) digestions were performed using an enzyme to protein ratio of 1:100 following manufacturer’s instructions. TFA at final concentration of 0.7% was added to the samples to stop the digestion and cleave RapiGest surfactant. Samples were filtered in stage tips, using solid-phase extraction with C18 stage tips, and eluted with 40% acetonitrile. Stage tips were assembled with InertSep RP-C18 resin (GL Sciences) and SDB-XC membrane (Empore, 3M) inside P200 pipette tips. The eluates were dried in a vacuum concentrator (Concentrator Plus, Eppendorf) and stored at -20°C until MS analysis [51].

LC-MS/MS analyses were performed in triplicates, using the chromatographic system nanoAcquity UPLC (Waters) coupled to a Synapt G2 HDMS mass spectrometer (Waters). Samples (10 μg) were loaded into a trap column (Acquity UPLC M-Class Symmetry C18 Trap Column, 100 Å, 5 μm, 300 μm x 25 mm, Waters) at 8 μL/min of deionized water, 5% acetonitrile, 0.1% formic acid for 5 min. Then, the mixture of trapped peptides was eluted in an analytical column (Acquity UPLC M-Class HSS T3 Column, 1.8 μm, 300 μm x 150 mm, Waters) with a gradient of 5–35% of phase B (0.1% formic acid in acetonitrile), phase A (0.1% formic acid in deionized water), over 60 min at a flow rate of 3 μL/min. MS data were acquired on data independent mode (DIA), using ion mobility separation HDMS^E^, in the m/z range of 50–2,000 and set up in resolution mode. Peptide ions were fragmented by collision-induced dissociation (CID), in which collision energies were alternated between low (4 eV) and high (ramped from 17 to 60 eV) for precursor ion and fragment ions, respectively, using scan time of 1.0 s. The ESI source was operated in the positive mode with a capillary voltage of 2.7 kV, block temperature of 100°C, and cone voltage of 40 V. The column temperature was set at 55°C and samples were maintained in autosampler at 10°C. For lock mass correction, a [Glu1]-Fibrinopeptide B solution (500 fmol/mL in 50% methanol, 0.1% formic acid; Peptide 2.0) was infused through the reference sprayer at 2 μL/min and sampled every 60 s for external calibration [51, 52]. The mass spectrometry data have been deposited to the ProteomeXchange Consortium via PRIDE repository [10.1093/nar/gky1106], with dataset identifier PXD034977.

Label-free quantification (LFQ) was performed in Progenesis QI for proteomics (NonLinear Dynamics, Newcastle, UK) as previously reported [52]. Briefly, the raw files were loaded in the software and samples were aligned based on precursor ion retention time of reference run, which was automatically selected. Default peak picking parameters were applied. The MS data were processed by the Apex3D module using a low energy threshold of 750 counts and high energy threshold of 50 counts. The MS/MS spectra were exported as.mgf file for protein identification in PEAKS Studio 7.5 (Bioinformatics Solution Inc.) and run against *Bothrops jararaca* sequences obtained from the National Center for Biotechnology Information (NCBI: http://www.ncbi.nlm.nih.gov/), including 86 annotated sequences from the *B*. *jararaca* genome (Bioproject PRJNA691605 [https://doi.org/10.1073/pnas.2015159118]), totalizing 437 sequences (downloaded on July 11, 2023) [53–55]. Search parameters were set as follows: mass tolerance of 10 ppm for precursor ions and 0.025 Da for fragment ions, up to two missed cleavage sites allowed for trypsin digestion, FDR of 1% at the peptide level, and minimum of 1 unique peptide per protein. Carbamidomethyl cysteine residues were selected as fixed modification, and methionine oxidation as variable modifications. The identification results were then reimported to Progenesis as a.pepXml file. Proteins were quantified by calculating the sum of all normalized abundances of assigned peptides corresponding to that protein.

### 2.7. Statistical analysis

The protein bands observed in the Western blotting experiment were quantified using ImageJ software. The acquired data was further analyzed and presented using GraphPad Prism 9.0.2 (GraphPad Prism) software. To evaluate the statistical significance of the assays involving pooled samples, a Brown-Forsythe one-way ANOVA was performed. The Pearson test was employed to assess the correlation between the results of individual samples and SVL (snout-vent length). Statistical significance was determined based on p-values less than 0.0001. The relative abundance, expressed as a percentage, was calculated using the raw values of the normalized abundance for each group. For the 2 sequences of γPLI class inhibitors, the raw values of the previously normalized abundance were averaged. The statistical analysis of the mass data between the groups was conducted using Two-Way ANOVA, comparing the means of each column (Group), followed by Tukey’s multiple comparisons test. Statistical significance was determined based on p-values less than 0.0001.

## 3. Results

### 3.1. Analysis of plasma-venom protein interactions

The ontogenetic and sexual variability of PLI in plasma composition of *B*. *jararaca* snakes were analyzed using ninety specimens **(Table 1).** These specimens were divided into six groups according to sex and age (neonates, youngs, and adults; female and male). It is important to note that sample pooling was conducted prior to analyses due to the limited amount of venom obtained from each neonate individual. This pooling strategy was necessary to ensure an adequate sample size for analysis. Samples from neonates were plasma pools from siblings of specimens that were 2 days old, young specimens ranged from 16 to 20 months old, and although we cannot report the age of adults, specimens that had been in captivity for more than 25 months were used. It is important to highlight that, despite the use of specimens from different regions (São Paulo, Santa Catarina, Minas Gerais, and Rio Grande do Sul), no relation was found between the results presented below and geographic variability (F (3,31) = 0.6332, *p* = 0.5992).

**Fig 2:**
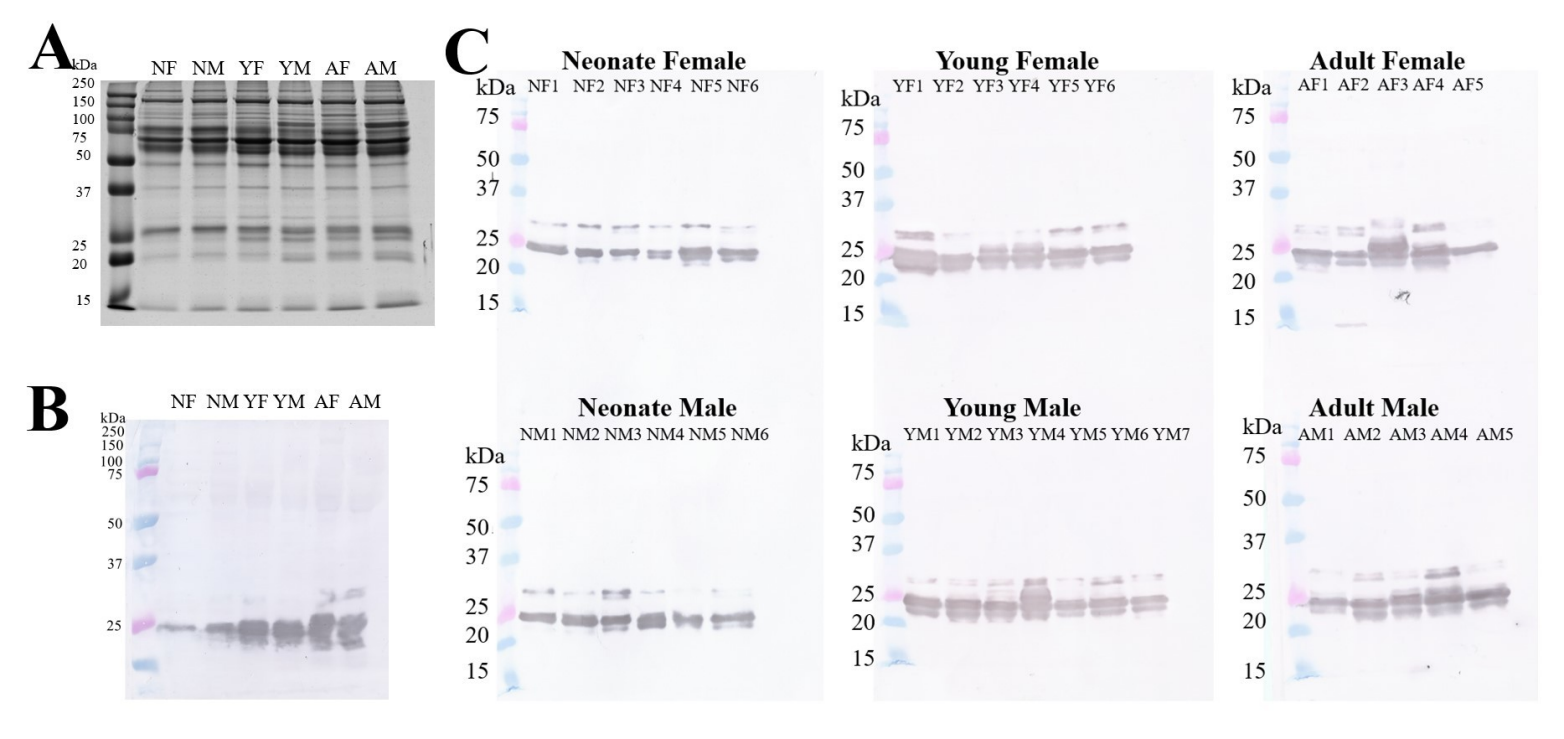
SDS-PAGE and Western blotting assay with *B*. *jararaca* venom. **(A)** SDS-PAGE 15% with 20 μg of each plasma pools samples. **(B)** Recognition of venom proteins by *B*. *jararaca* pooled plasma. Twenty μg of plasma proteins were applied on SDS-PAGE. After transferring the proteins to a PVDF membrane, it was sensitized with 40 μg of *B*. *jararaca* venom, incubated with antibothropic serum (1:1,000), and then incubated with horse anti-IgG conjugated with peroxidase (1:10,000). The reaction was developed using a chromogenic substrate (3,3-diaminobenzidine (tetrahydrochloride)). **(C)** Recognition of venom proteins by individual plasma samples. Twenty μg of plasma proteins were applied on SDS-PAGE. After transferring the proteins to a PVDF membrane, it was sensitized with 40 μg of *B*. *jararaca* venom, incubated with antibothropic antibody (1:1,000), and then incubated with horse anti-IgG conjugated with peroxidase (1:10,000). Finally, the reaction was developed using a chromogenic substrate (3,3’diaminobenzidine (tetrahydrochloride)). Neonate female (NF), neonate male (NM), young female (YF), young male (YM), adult female (AF) and adult male (AM).

First, the electrophoretic profiles of the plasma showed a complex composition of proteins **(Fig 2A)**, with a greater number of bands with high molecular mass between 50 and 200 kDa, and a few bands with lower molecular mass around 25 kDa. In order to investigate which of these bands may interact with *B*. *jararaca* venom proteins, a recognition analysis by Western blotting was performed, revealing a lower number of bands, which were predominantly present around 25 kDa **(Fig 2B and 2C).** Analysis of the pooled samples **(Fig 2B)** showed a marked increase in band number and intensity according to age in both sexes, whereas this pattern was absent in individual analysis (months vs female: r = 0.2133, R^2^ = 0.04863, *p* = 0.3964; months vs male: r = 0.2038, R^2^ = 0.04152, *p* = 0.4174) **(Fig 2C).** In a detailed evaluation of the detection profile of the pooled samples, in addition to the 25 kDa band in all groups, the presence of a ~24 kDa band in the young group and two bands at ~24 kDa and ~30 kDa, respectively, in the adult group is noteworthy **(Fig 2B and 2C).** On the other hand, in the profile of individual samples, the ~30 kDa band is also seen in some neonate and young samples **(Fig 2C).**

### 3.2. Quantification of plasma-venom protein interactions

**Fig 3:**
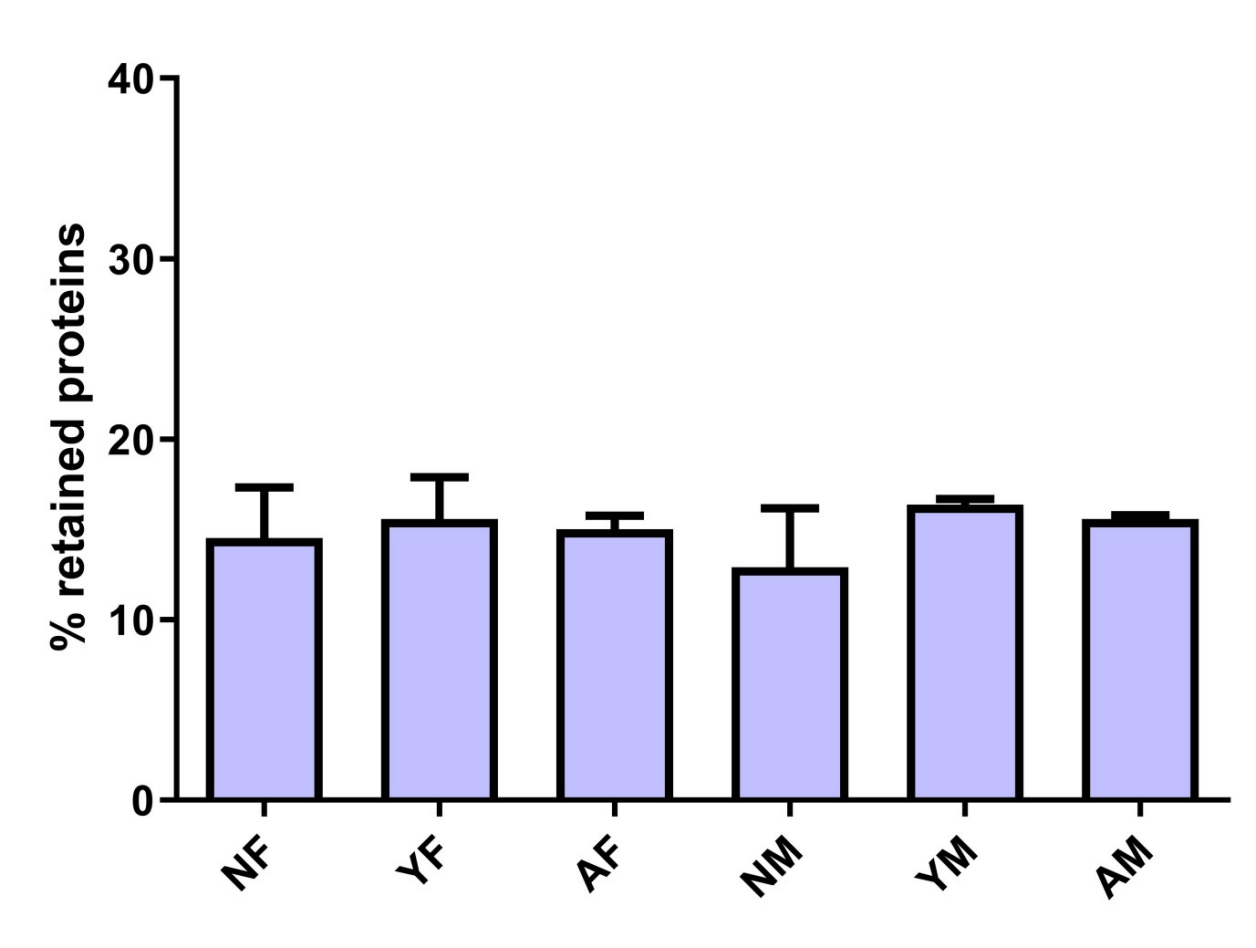
Percentage of proteins retained and eluted by affinity chromatography. Samples from the plasma pools of female neonates (NF), male neonates (NM), young females (JF), young males (JM), adult females (AF), and adult males (AM) were applied to an affinity column containing *B*. *jararaca* venom coupled to the CNBr-activated Sepharose Fast-Flow resin. Plasma proteins adsorbed to the resin-coupled *B*. *jararaca* venom proteins were eluted using glycine and the replicated of % retained protein was plotted as mean SD.

After confirming the plasma-venom interactions qualitatively, plasma proteins that had affinity for venom toxins were isolated using affinity chromatography, in which *B*. *jararaca* venom was coupled to a resin. This analysis was performed with pooled plasma due to the amount of sample required. After quantification of the total protein eluted from the column **(Fig 3)**, we observed that all plasma samples assayed presented ~15% of proteins that could interact with the *B*. *jararaca* venom proteins, and that there was no statistically significant difference between the groups (F (5,29) = 2.254, *p* = 0,0755).

### 3.3. Detection of γBjPLI presence in snake plasma

**Fig 4:**
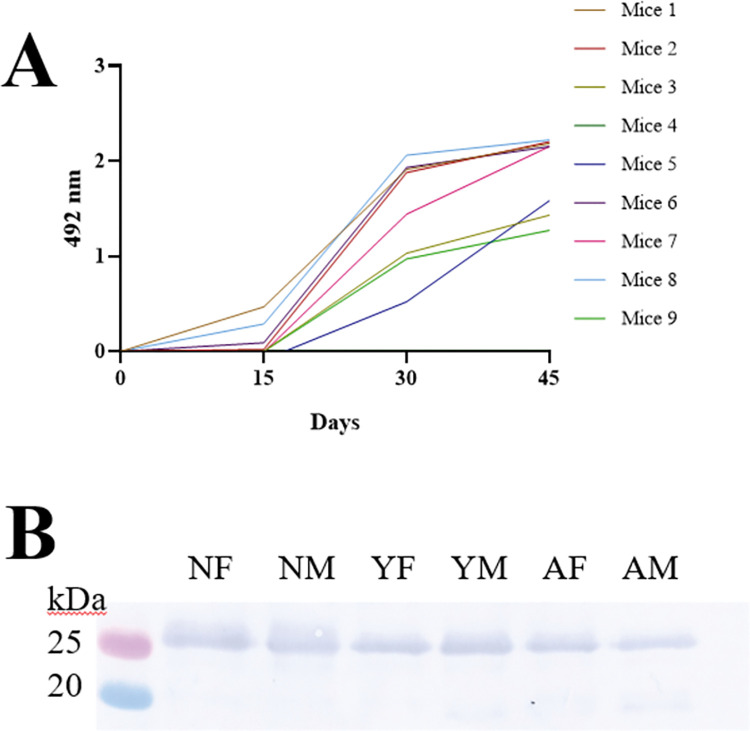
Imunization and immunorecognition assay with anti-γBjPLI. **(A)** Immune response profile of mice immunized with γBjPLI. **(B)** Immunorecognition of plasma pools. Twenty μg of plasma proteins were applied on SDS-PAGE. After transferring the proteins to a PVDF membrane, it was incubated with anti- γBjPLI (1:1,000), followed by the incubation with anti-mouse IgG peroxidase-conjugated (1:10,000), and the reaction was developed using a chromogenic substrate (3,3-diaminobenzidine (tetrahydrochloride)). Neonate female (NF), neonate male (NM), young female (YF), young male (YM), adult female (AF) and adult male (AM).

Our next step was to perform a more detailed analysis, in which the preparation of antibodies against γBjPLI (anti-γBjPLI) was performed. This allowed us to identify the presence of this inhibitor in each sample. Antibody was raised in 9 mice using the isolated γBjPLI. The ideal antibody titration of each mouse was achieved after the third immunization **(Fig 4A)**, so the pool of 9 mice serum was prepared (titration = 10.6 x 10^3^) and subsequent experiments could be performed. Immunoblotting by Western blotting confirmed the presence of γBjPLI in all pooled samples **(Fig 4B)**.

### 3.4. Proteomic analysis of plasmas of snakes by mass spectrometry

To identify the inhibitors more precisely, pooled NF, NM, YF, YM, AF, and AM plasma samples were subjected to shotgun proteomics. Due to the small number of snake plasma proteins deposited in the database, only five plasma proteins were identified **(Table 2)**. Among the identified proteins, four were identified as PLI-family proteins, one was identified as antihemorrhagic protein.

**Table 2:** List of proteins identified by mass spectrometry in the plasma of *Bothrops jararaca* snake.

Accession	PLI Class	Mass (kDa)	Unique peptides	Description
B1A4N8.1	αPLI	18.3	31	Alpha Phospholipase A2 inhibitor—*Bothrops jararaca*
QBG82015.1	βPLI	37.3	6	Beta Phospholipase A2 inhibitor—*Bothrops jararaca*
ABV91330.1	γPLI	22.0	1	Gamma Phospholipase A2 inhibitor—*Bothrops jararaca*
ABV91331.1	γPLI	22.1	2	Gamma Phospholipase A2 inhibitor—*Bothrops jararaca*
Q9DGI0.1		38.7	50	Antihemorrhagic factor BJ46a - *Bothrops jararaca*

Regarding the PLIs, the proteomic approach utilized herein identified the inhibitors of the three classes (αPLI, βPLI, and γPLI). Although the γ-type inhibitor had already been isolated, to the best of our knowledge, this is the first time that α- and β-type PLIs have been identified in the plasma of *B*. *jararaca* snakes by proteomic analysis. One sequence described as αPLI was identified, namely B1A4N8 with approximately 18 kDa. Only one protein of 37 kDa was identified, which refers to the PLI class β and corresponds to accession number QBG82015.1. As for the γ-inhibitor, two sequences were identified in the database, ABV91330.1 and ABV91331.1, with a molecular mass of 22.0 kDa and 22.1 kDa, respectively. Another group of proteins that have been described in the literature with a protective function related to the activities of toxins are the antihemorrhagic factors [29, 53]. These antihemorrhagic factors, present in the plasma of venomous animals, are also called metalloprotease inhibitors. Our results of proteomic analysis of the plasmas showed sequences corresponding to these antihemorrhagic factors, with molecular mass of 38 kDa and accession number Q9DGI0 (BJ46a).

**Fig 5:**
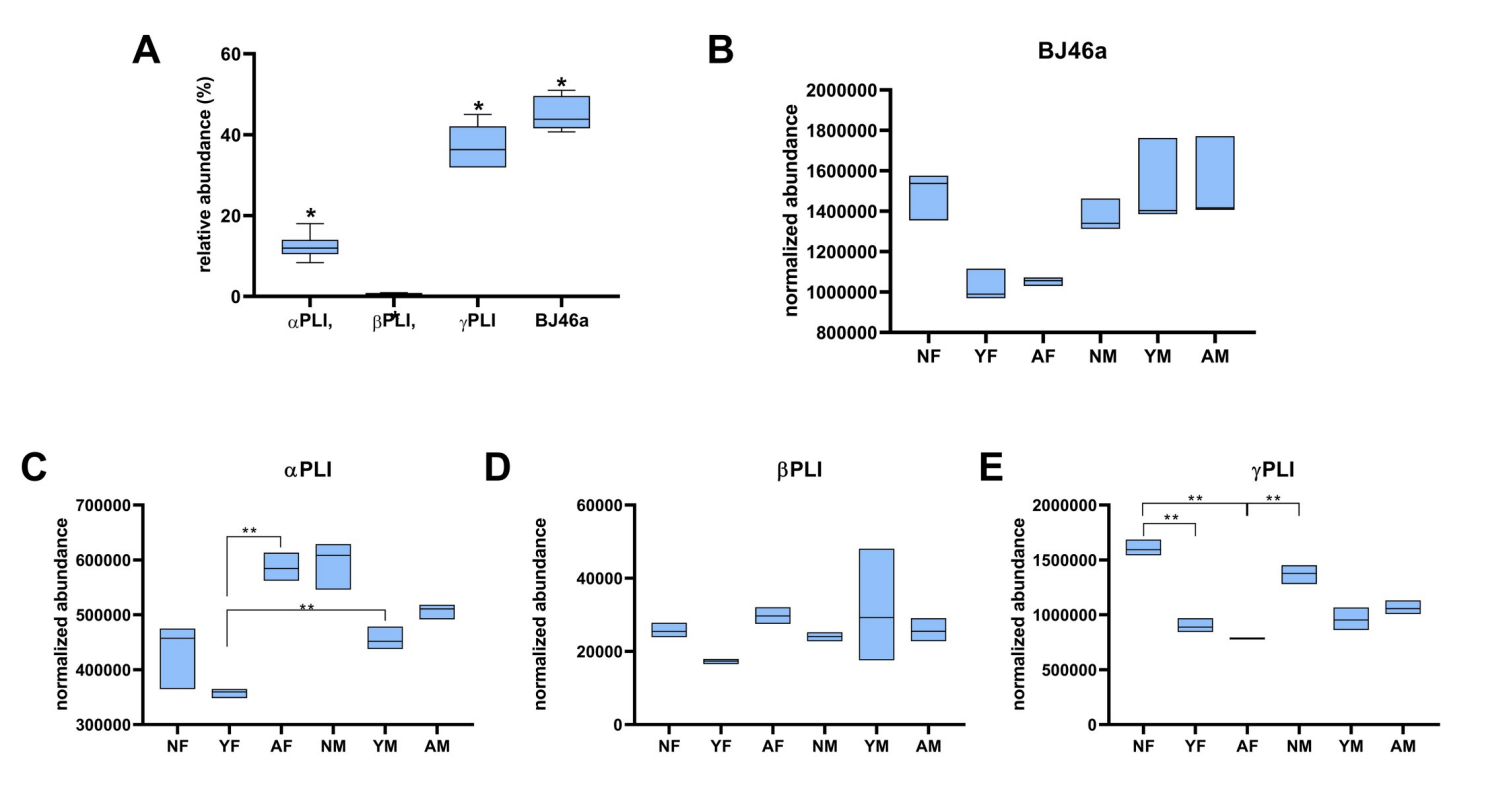
Relative abundance (%) and normalized abundance of PLI classes and BJ46a in plasma of snakes of the species *B*. *jararaca* identified by mass spectrometry. All plasma pools were subjected to LC-MS/MS, and proteins were identified, quantified, and assigned to each protein family expressed**. (A)** PLI classes relative abundance. (**B)** BJ46a normalized abundance. **(C)** αPLI normalized abundance. **(D)** βPLI normalized abundance. **(E)** γPLI normalized e abundance. The floating bars represent the minimum and maximum values and the central line the average value. *Indicates a statistically significant difference compared with others two groups (p<0.0001, two-way ANOVA, Tukey as a post hoc test). **Indicates a statistically significant difference (*p*<0.0001, two-way ANOVA with Tukey as a post hoc test).

The mass spectrometry results showed a statistical difference in the proportion of each class (*p <* 0.0001), with BJ46a (45%) being the most abundant, followed by γPLI (37%), αPLI (12%) and βPLI (0.6%) **(Fig 5)**.

When the normalized abundance of each identified protein is analyzed separately, a diversity of averages can be observed. However, only the αPLI and γPLI inhibitors showed significant differences (p < 0.0001) between the groups **(Fig 5C and 5E)**. αPLI exhibited an increase in AF when compared to YF, and YF was lower than YM. On the other hand, γPLI showed a tendency of decrease in abundance with increasing age in females and males.

## 4. Discussion

In this work, using the techniques of SDS-PAGE, Western blotting, and affinity chromatography, it was possible to observe the presence of proteins in the plasma of young and adult snakes interacting with proteins from the venom of *B*. *jararaca*, corroborating previous studies [31, 33]. Moreover, the presence of these proteins was detected for the first time in 2-day-old neonates. Mass spectrometry analyses detected the presence of PLA_2_ inhibitor proteins of the αPLI, βPLI, and γPLI classes, and of antihemorrhagic factor described as metalloprotease inhibitors. The presence of γBjPLI was also confirmed in all plasma samples analyzed by Western blotting.

### 4.1. Analysis of plasma-venom protein interactions

Using antivenom techniques described by Calvete et al. (2011) [56] and based on affinity chromatographies, Gibbs et al. (2020) [21] isolated and identified 45 types of plasma proteins with probable resistance function present in California ground squirrels (*Otospermophilus beecheyi*) naturally preyed by the venomous snake *Crotalus oreganus oreganus*, with disintegrins, metalloproteases, and PLA_2_ from venom as the main targets of the isolated proteins [21, 49]. In snakes, Gomes et al. (2017) [33] analyzed the liver transcriptome of *B*. *jararaca*, and identified transcripts of γPLI, BJ46a-like, inter-alpha-trypsin inhibitor, and C1 protease inhibitor. Moreover, Morais-Zani et al. (2013) [31] described the presence of complement system proteins, in addition to BJ46a and γPLI in *B*. *jararaca* plasma [19, 31–33]. In this study, an increase in the intensity and number of plasma protein bands that interacted with the venom toxins were observed by Western blotting **(Fig 2B)** only at 25 kDa. This result suggests that only γPLI isoforms were detected. However, mass spectrometry analysis **(Table 2)** identified other proteins, including αPLI, βPLI, and BJ46a.

### 4.2. Detection of γBjPLI presence in snake plasma

To clarify which protein bands corresponded to γBjPLI, the samples were submitted to Western blotting using anti-γBjPLI antibodies produced in BALB/c mice. The results of Western blotting show that the pattern of bands detected by anti-γBjPLI and by *B*. *jararaca* venom were not identical. In the first case **(Fig 4B)**, only a single band of 25 kDa was detected, with no differences in intensity in the groups studied (neonates, young, and adults, females and males), whereas in the second case, the intensity and number of bands increased with age **(Fig 2B)**, suggesting ontogenetic variability in the composition of inhibitors in these animals. Spots between 20 kDa and 25 kDa, identified as PLIs in another proteomic study, can be associated with the bands detected in the Western blotting of *B*. *jararaca* venom presented in this work **(Fig 2B)**, supporting the hypothesis that there are different isoforms of PLIs, which increasing concentration during ontogenetic development [31]. On the other hand, it was not possible to correlate the band occurring just above 25 kDa with any plasma protein already described in the literature, which requires further investigation.

### 4.3. Proteomic analysis of plasmas of snakes by mass spectrometry

Although the immunorecognition by Western blotting **(Fig 2B)** did not detect α-class (18 kDa) and β-class (37 kDa) inhibitors, the results of the mass spectrometry analysis **(Table 2)** showed that the three classes of PLI were present in the plasma of the *B*. *jararaca* snakes. In the case of βPLI, this could be due to the low abundance of these proteins in plasma compared with the α-class and γ-class inhibitors, as shown in **Fig 5**. Liver transcriptome analysis revealed a 30-fold increase in the expression of γPLIs in adults compared to young, while proteomics described in literature and in this work is the opposite, with a greater amount in young than in adults, which accounted for 70% and 30%, respectively, of the total number of proteins identified [31, 33].

The difference in protein expression between young and adult samples found in the transcriptome work may also be related to post-transcriptional factors, as protein expression often needs to be regulated so that they are not constantly expressed, which ensures the plasticity of organisms, highlighting the significance that transcriptional data must be corroborated through proteomic analysis, otherwise it may result in erroneous inferences [33]. The process of gene expression regulation involves complex interactions between transcription factors, enhancers, and other regulatory elements that control the activation or repression of specific genes. These regulatory elements can be influenced by environmental factors, signaling pathways, and developmental cues, adding another layer of complexity to the regulation of gene expression. Similarly, the translation of proteins from mRNA is subject to multiple levels of regulation. This includes the availability of ribosomes, initiation factors, and regulatory sequences within the mRNA itself. Post-translational modifications, such as proteolytic cleavage or protein folding, can further modify the final composition and function of proteins. The interplay of these regulatory mechanisms contributes to the extensive variation in venom composition observed among snake species. Even closely related species can exhibit distinct venom profiles due to differences in the regulation of gene expression and protein translation [57–59].

The presence of PLI classes and antihemorrhagic factors in newborns is probably due to the need to protect themselves from both self-envenomation and snakebite from siblings, since they are viviparous snakes and up to 5 to 16 developed newborns can be born in each litter [60, 61]. In snakes, females are known to be heterogametic (ZW) while males are homogametic (ZZ). However, it should be noted that sex chromosomes can have diverse effects on individual physiology and behavior. Some species exhibit sex-specific differences in hormonal regulation and plasma levels of certain compounds, which can impact reproductive stages and behaviors. Moreover, sex-specific gene expression and the presence of sex-linked genes on sex chromosomes may contribute to variations in physiological processes [62]. According to Passos (2018) [63], males typically reach sexual maturity around 13 months of age, regardless of feeding type, whereas females become sexually mature between 17 and 22 months, depending on the feeding type. However, other authors suggest that female sexual maturation occurs around 24 months [63, 64].

Therefore, variations in αPLI levels in the plasma of *B*. *jararaca* may be influenced by additional biological factors associated with individual maturation, given that the αPLI levels in young females were found to be lower than those in young males. In this study, the group of young females, up to 20 months old, may not have attained full maturity, whereas the males, aged over 16 months, were likely already mature.

## 5. Conclusion

In summary, the present work demonstrated that 9.63–17.9% of *B*. *jararaca* plasma proteins can recognize the venom proteins of the same species. The presence of different endogenous inhibitors and, more specifically, different isoforms of PLI in newborn snakes of *B*. *jararaca* were identified. αPLI and βPLI were detected for the first time in plasma of newborn, young, and adult of *B*. *jararaca*. In addition, BJ46a (45%) and γPLI (37%) were the most abundant inhibitors identified, followed by αPLI (12%) and βPLI (0.6%). The findings presented in this study on the ontogeny of *B*. *jararac*a plasma inhibitor composition hold significant implications in the broader context of venom biology and evolution. The identification of different endogenous inhibitors, particularly various isoforms of PLI, in newborn snakes of *B*. *jararaca* provides valuable insights into the mechanisms of natural resistance to envenomation. The detection of αPLI and βPLI for the first time in the plasma of newborn, young, and adult snakes indicates a nuanced complexity in the composition of these inhibitory compounds across different developmental stages. The observed lack of significant ontogenetic and sexual correlation in the presence of these inhibitors suggests a sophisticated and finely tuned regulatory system that might not solely be driven by age or gender factors. Additionally, the abundance of BJ46a and γPLI as the most prevalent inhibitors in different developmental stages underscores their potential role in counteracting the effects of self-envenomation from birth. These findings contribute to our understanding of the intricate interplay between snake venoms and endogenous inhibitors, shedding light on the evolutionary adaptations that may have shaped the venom resistance mechanisms in *B*. *jararaca* and potentially other snake species. The identification and characterization of these inhibitors provide a foundation for further exploration into the evolutionary arms race between venomous snakes and their prey, offering insights that could inform antivenom development and strategies for mitigating the impact of snakebites.

## Supporting information

S1 Raw images This section includes the unaltered original images utilized in this study, presented without any digital post-processing. (PDF)

S1 Raw data This section encompasses the unmodified primary data utilized in this research, presented in its original state without any modifications. (XLSX)
