# Supplementary material for: Proteomics and life-history variability of Endogenous Phospholipases A2 Inhibitors (PLIs) in Bothrops jararaca plasma
Source: PLoS One. 2024 Feb 6;19(2):e0295806. doi: 10.1371/journal.pone.0295806 (PMC10846723; doi:10.1371/journal.pone.0295806)

Fig 2A. SDS-PAGE scanned using Image Scanner III (GE Healthcare).

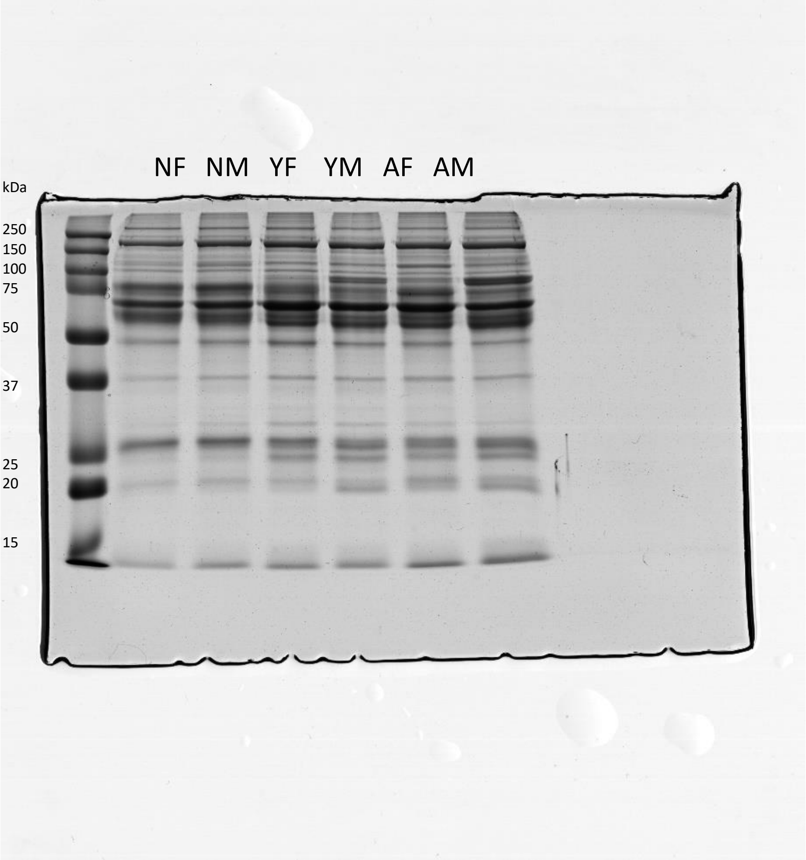

Fig 2B. Western blotting scanned using Image Scanner III (GE Healthcare).

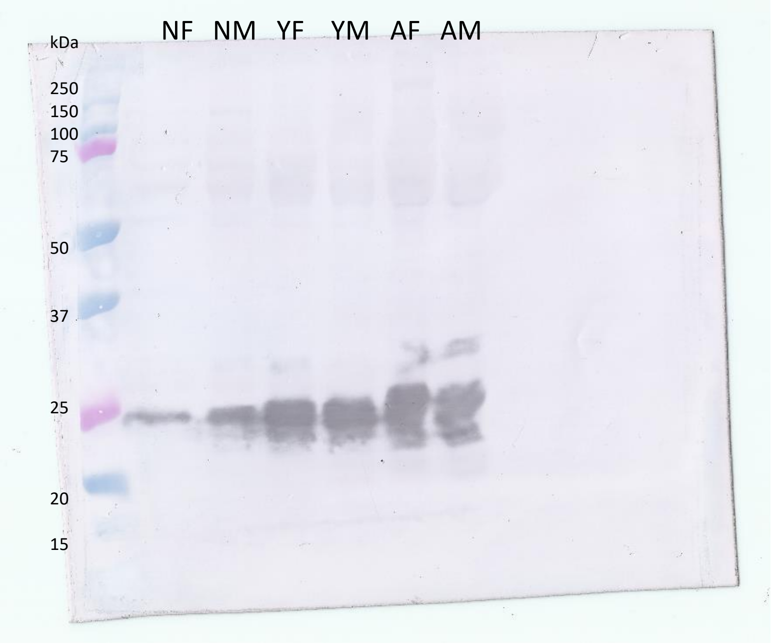

Fig 2C. Western blotting scanned using Image Scanner III (GE Healthcare).

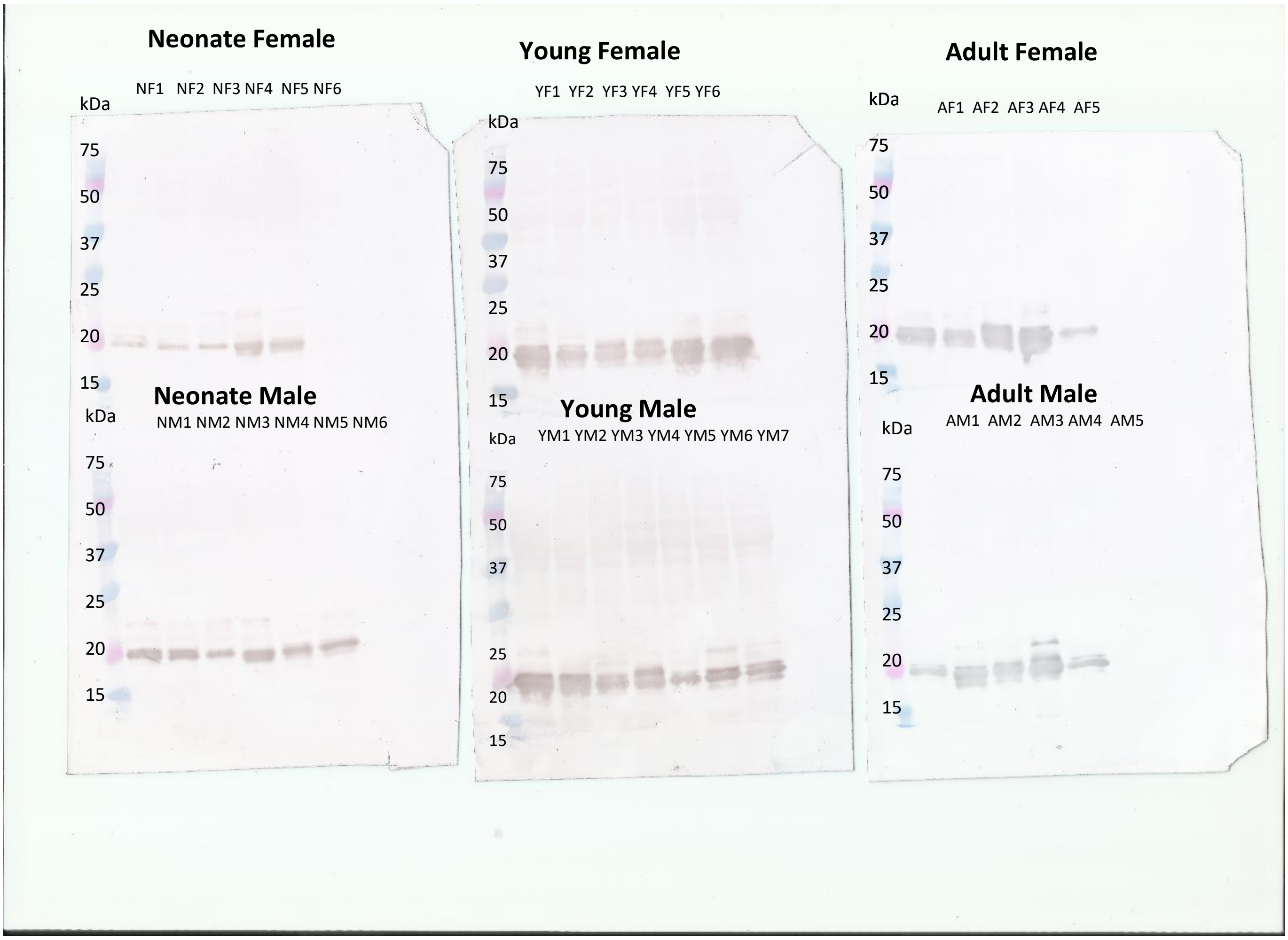

Fig 4B. Western blotting scanned using Image Scanner III (GE Healthcare).

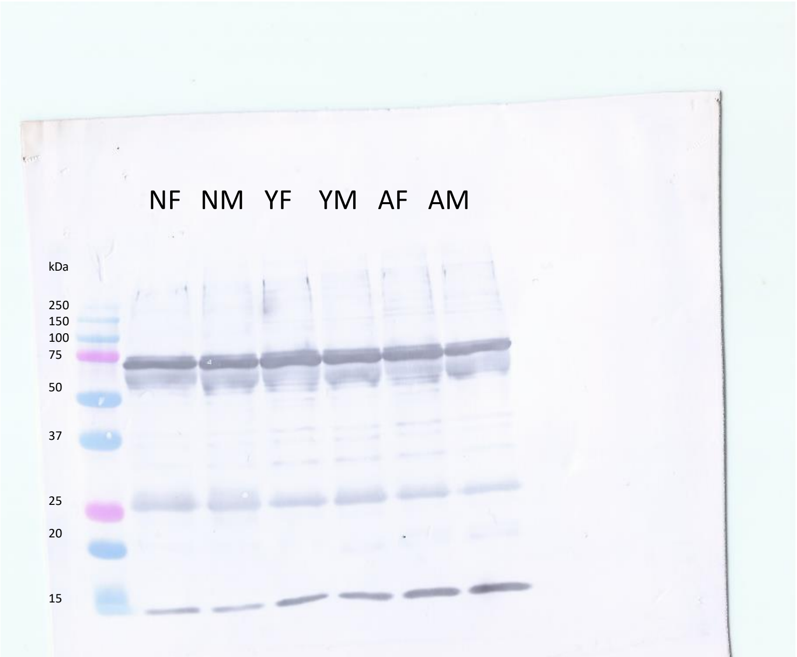

Supplement: S1 Raw images — (PDF) [file pone.0295806.s001.pdf]
